# Supplementary material for: Development and validation of a data quality index for forensic documentation of sexual and gender-based violence in Kenya
Source: PLoS One. 2022 Jan 27;17(1):e0262297. doi: 10.1371/journal.pone.0262297 (PMC8794179; doi:10.1371/journal.pone.0262297)
Supplement: Attachment — Submitted filename: Response_to_Reviewers .docx [file pone.0262297.s002.docx]

**RESPONSE TO REVIEWERS**

Ms. Ref. No.: PONE-D-21-17812

Article Title: Development and validation of a data quality index for forensic documentation of sexual and gender-based violence in Kenya

| Reviewer #1  All seem to be in order. There are few errors in Reference list. Reference No.5: "Full Article" is indicated as name of author. Please find real authors for accurate referencing. Nu6: It is unusual to start with the year. Number. 12 does not seem to be in sync with other references, revise referencing style. | Author Responses  Thank you very much for your review and we agree with your suggestion. We have reviewed all references and ensured they are now accurate per the Vancouver reference style. |
| --- | --- |
| Editor Comments | Author Responses |
| Thank you for this important manuscript. I apologize about the delay in review time. I tried repeatedly to get more than one reviewer with no success. Both myself and Reviewer #1 agree that this is a very well-written and important manuscript. Reviewer #1 has recommended some minor edits to the reference list (which I agree with) and should be undertaken before publication. Thank you for your important work. | Thank you for your comments and we appreciate your efforts and enthusiasm. We have ensured all references are now in accordance with the Author Guidelines and accurately reported. |
